# The Prognostic Nutritional Index as a Lifeline for Cardiovascular Health in Individuals With Insomnia: A Prospective Cohort Study

**DOI:** 10.1002/hsr2.72920

**Published:** 2026-08-09

**Authors:** Zihao Liu, Linguo Gu

**Affiliations:** ^1^ Endoscopy Center, The Affiliated Cancer Hospital of Xiangya School of Medicine Central South University/Hunan Cancer Hospital Changsha Hunan China; ^2^ Department of Thoracic Surgery, The Second Xiangya Hospital Central South University Changsha Hunan China; ^3^ Thoracic Surgery Research Laboratory, The Second Xiangya Hospital Central South University Changsha Hunan China; ^4^ Hunan Key Laboratory of Early Diagnosis and Precise Treatment of Lung Cancer, The Second Xiangya Hospital Central South University Changsha Hunan China

**Keywords:** cardiovascular risk, insomnia, mortality, NHANES, prognostic nutritional index

## Abstract

**Background and Aims:**

This study aimed to examine the relationship between the prognostic nutritional index (PNI) and both all‐cause and cardiovascular mortality among individuals diagnosed with insomnia.

**Methods:**

A total of 3161 participants from the NHANES database were analyzed, stratified by the severity of insomnia (mild, moderate, severe). Weighted Cox regression models were employed to evaluate the associations between PNI and mortality, while subgroup and interaction analyses were conducted to assess consistency across various demographic groups. Kaplan–Meier survival curves were utilized to compare survival rates across PNI quartiles, and Receiver Operating Characteristic (ROC) curves were used to evaluate the predictive accuracy of PNI. Additionally, Restricted Cubic Spline (RCS) analysis was performed to identify safety thresholds for PNI.

**Results:**

Among the participants, 16% were classified as having severe insomnia. A higher PNI, within a specific range, was found to be protective against cardiovascular mortality (Hazard Ratio [HR] = 0.24, 95% Confidence Interval [CI]: 0.14–0.41, *p* < 0.05) and all‐cause mortality (HR = 0.37, 95% CI: 0.27–0.50, *p* < 0.001), after adjusting for confounding factors such as age, sex, race, and education. The RCS analysis revealed a nonlinear relationship, indicating that a PNI below 51.9 was associated with an increased risk of cardiovascular mortality.

**Conclusion:**

An elevated PNI, within a defined range, is associated with reduced mortality risks in patients with insomnia, whereas PNI levels below 53.35 for all‐cause mortality and 51.9 for cardiovascular mortality are associated with heightened risk. PNI may serve as a valuable prognostic tool in this population.

**Clinical Trial Registration:**

NA.

**Abbreviations**
ALT: alanine aminotransferase
AST: aspartate aminotransferase
BMI: body mass index
CI: confidence interval
CVD: cardiovascular events
DBP: diastolic blood pressure
HR: hazard ratio
NHANES: National Health and Nutrition Examination Survey
PIR: poverty income ratio
PNI: prognostic nutritional index
RCS: restricted cubic splines
ROC: receiver operating characteristics
SBP: systolic blood pressure

## 1 Introduction

Insomnia, a condition self‐reported by patients, is characterized by an inability to initiate or maintain sleep, manifesting as frequent nocturnal awakenings, difficulty resuming sleep, or premature morning awakenings without the ability to fall back asleep [1]. Insomnia, as the most prevalent sleep disorder, represents a substantial burden on the healthcare system [2]. From 1993 to 2015, the prevalence of insomnia diagnoses during office visits in the United States rose 11 times, from 800,000 to 9.4 million [3]. Insomnia is closely related to various factors such as age, gender, genetic predisposition, life stress, and medication use [4]. On one hand, insomnia exacerbates the risk of developing hypertension, diabetes, and cardiovascular diseases [5, 6, 7, 8, 9]. On the other hand, insomnia further intensifies the risk of mortality, particularly with respect to cardiovascular‐related deaths [10, 11, 12]. Existing research indicates that individuals with insomnia have a 29% higher risk of cardiovascular mortality compared to those without insomnia [13]. Studies have shown that malnutrition can lead to increased oxidative stress and alterations in calcium‐handling proteins, thereby contributing to myocardial dysfunction. For example, in a malnutrition model induced by a low‐protein diet, myocardial oxidative stress levels are significantly elevated, and the expression of calcium‐handling proteins such as sarcoplasmic reticulum Ca^2+^‐ATPase (SERCA) is reduced—both of which are associated with decreased myocardial contractile function [14]. Research indicates a certain association between insomnia and malnutrition [15, 16]. However, whether nutritional status correlates with the occurrence of cardiovascular events remains underexplored. A study investigating malnutrition in patients with obstructive sleep apnea (OSA) and acute coronary syndrome (ACS) found that malnutrition independently correlates with the risk of cardiovascular events, especially among patients comorbid with OSA [17]. This suggests that malnutrition may exacerbate cardiovascular risk in specific populations with sleep disorders. After alleviating insomnia symptoms, the risk of cardiovascular mortality decreases, highlighting the substantial importance of focusing on sleep treatment and reducing the prevalence of insomnia [18, 19, 20, 21].

In addressing insomnia treatment, a considerable body of research has concentrated on cognitive‐behavioral therapy, which remains the gold standard in managing this condition [22]. Moreover, alternative interventions such as acupuncture, physical exercise, and pharmacotherapy have demonstrated efficacy in alleviating symptoms of insomnia [23, 24]. However, recent studies have indicated that nutritional intake is a significant factor influencing sleep. Both excessive and insufficient protein consumption in the diet can lead to a decline in sleep quality [25]. Certain amino acids in the diet, particularly tryptophan, are pivotal in influencing sleep. The metabolites of tryptophan, melatonin, and serotonin, are critical chemical substances in the regulation of sleep [26]. Additionally, the metabolism of carbohydrates and fatty acids is closely linked to sleep quality [26, 27]. These studies indicate that both malnutrition and overnutrition can adversely affect sleep quality, potentially leading to insomnia.

The prognostic nutritional index (PNI) is a newly proposed nutrition‐related index, which offers a significant advantage over the separate assessment of albumin and total lymphocyte count as an indicator of nutritional status [28, 29, 30]. PNI can be easily calculated using both albumin concentration and total lymphocyte count. The PNI has demonstrated strong prognostic value across diverse populations [31]. Several intriguing studies have discovered that individuals with a lower PNI face a significantly higher risk of both cardiovascular and all‐cause mortality [32, 33, 34]. A cohort study involving patients with chronic kidney disease revealed that those with higher PNI levels had approximately a 31% reduced risk of cardiovascular mortality compared to those with lower PNI levels [35]. These findings suggest that the PNI serves as an independent protective factor against cardiovascular mortality, with lower PNI levels correlating with an increased risk of cardiovascular death. However, to date, there is no literature reporting on whether PNI is associated with the incidence of cardiovascular mortality in individuals with insomnia.

As mentioned above, while the associations between nutrition, insomnia, and cardiovascular events have been well documented in numerous studies, the specific role of the PNI in populations with insomnia remains unreported in the existing literature. To investigate whether PNI levels are associated with the risk of cardiovascular mortality and all‐cause mortality in individuals with insomnia, we conducted this study using the National Health and Nutrition Examination Survey (NHANES) public database. This study will lay a solid foundation for future assessments of nutritional status and cardiovascular mortality risk in individuals with insomnia.

## 2 Materials and Methods

### 2.1 Participants and Study Design

The National Health and Nutrition Examination Survey (NHANES, www.cdc.gov/nchs/nhanes/index.htm) is a comprehensive program conducted by the National Center for Health Statistics (NCHS), part of the Centers for Disease Control and Prevention (CDC). Initiated in the early 1960s, NHANES is designed to assess the health and nutritional status of adults and children in the United States. This program employs a combination of interviews, physical examinations, and laboratory tests to collect data on a wide range of health indicators. NHANES is distinguished by its nationally representative sample, which allows for the generalization of findings to the entire U.S. population. The survey's rigorous methodology includes complex, multistage probability sampling, ensuring that diverse demographic groups are adequately represented. Data collected through NHANES include critical health measures such as anthropometric data, dietary intake, chronic disease prevalence, and biochemical markers. Each respondent in the NHANES survey provides informed consent prior to participation, ensuring ethical standards are upheld. The data generated are publicly available and freely accessible, contributing to open and transparent research practices.

**Figure 1:**
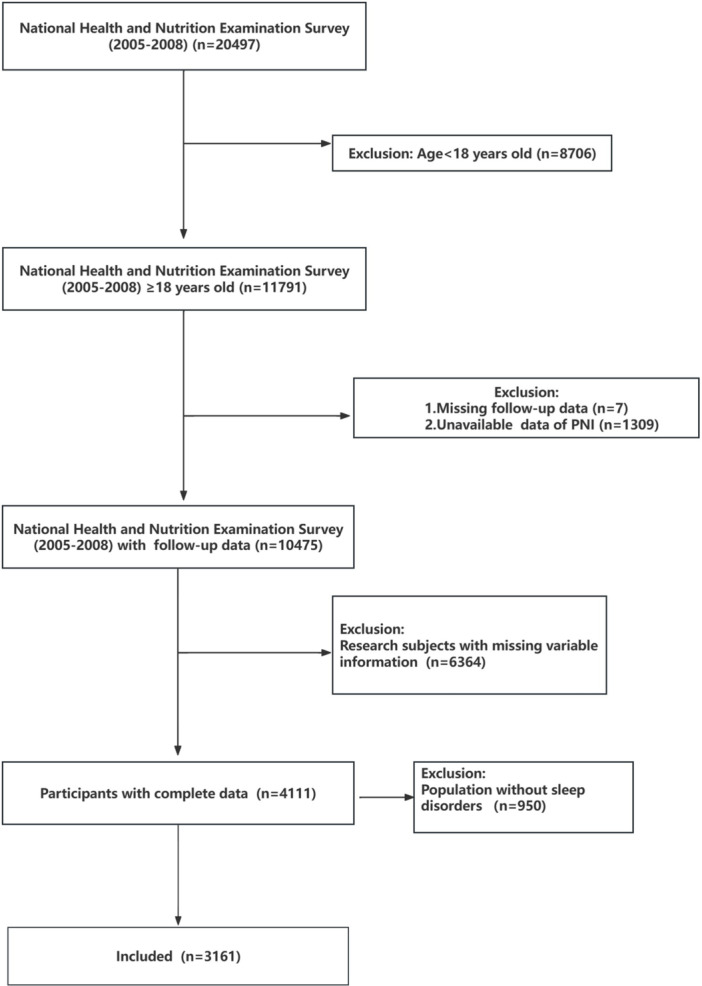
The research flow chart. The data were sourced from the NHANES database for the years 2005–2008, encompassing a total of 20,497 participants. Each participant has a unique SEQN number. Using this unique SEQN number, demographic data, physical examinations, laboratory tests, and questionnaire responses were compiled and merged.

In this study, we utilized data from the NHANES database spanning the years from 2005 to 2008. These specific years were selected because they uniquely included information from sleep assessment questionnaires. A total of 20,497 respondents participated in the survey during this period, providing a robust dataset for analysis. We initially excluded individuals under the age of 18 (*n* = 8706), as most information for this age group is restricted due to privacy regulations and therefore not available for analysis. Next, we excluded a total of 1316 respondents from the study due to missing data on survival follow‐up or the core exposure variable, PNI. Subsequently, we further excluded 6364 individuals from the study due to missing covariate information. Finally, based on the criteria for diagnosing insomnia, we excluded 950 individuals who did not have insomnia, resulting in a final cohort of 3161 patients with insomnia (Figure 1).

### 2.2 Exposure Variable

Serum albumin measurements were obtained from standard biochemical assays conducted in the laboratory, while lymphocyte counts were derived from the results of the complete blood count with 5‐part differential in the whole blood performed in the laboratory. PNI has been widely applied in research across various fields, including oncology, chronic kidney disease, post–coronary artery bypass grafting, and respiratory diseases [36, 37]. Building on previous studies, the formula for calculating PNI is provided below [29, 38]:

$\mathrm{PNI}=\mathrm{serum}\;\mathrm{albumin}\;(g/L)+5\times \mathrm{lymphocyte}\;\mathrm{count}\;(10^{9}/L)$.

### 2.3 Covariates

The covariates include demographic information (such as age, gender, race, education level, and the ratio of family income to poverty [PIR]), physical examination data (measurements of BMI and blood pressure), laboratory test results (serum creatinine, transaminases [ALT, AST], bilirubin, triglycerides [TG], total cholesterol [TC], fasting blood glucose and glycated hemoglobin, and serum cotinine levels), and questionnaire responses (smoking, alcohol consumption, presence of hypertension or diabetes, and sleep‐related questionnaires). Serum cotinine levels are used to assess patients' smoking status; the more frequent the smoking, the higher the serum cotinine levels [39, 40].

Hypertension is defined as follows: (1) having been diagnosed with hypertension by different physicians on at least two occasions, (2) systolic blood pressure > 140 mm Hg and/or diastolic blood pressure > 90 mm Hg measured on three separate occasions on different days, and (3) currently taking antihypertensive medication during follow‐up. Meeting any one of these three criteria qualifies as a diagnosis of hypertension [41]. Diabetes is diagnosed based on the following criteria: (1) being informed by two or more physicians of having diabetes, (2) fasting blood glucose > 7.0 mmol/L or glycated hemoglobin > 6.5%, or (3) current use of insulin or oral hypoglycemic agents to control blood sugar levels. Meeting any one of these three criteria qualifies as a diagnosis of diabetes [42]. Exposure information related to alcohol and tobacco was obtained from the questionnaire, and the determination of smoking and drinking status was based on methods reported in previous literature [43].

### 2.4 Assessment of Insomnia

In the NHANES questionnaire, the assessment of insomnia among participants is based on three core questions. These items capture key insomnia symptoms—including difficulty initiating sleep, difficulty maintaining sleep, and early morning awakening—along with their frequency and duration, which aligns with the clinical diagnostic criteria outlined in the Diagnostic and Statistical Manual of Mental Disorders, Fifth Edition (DSM‐5). However, given the absence of diagnostic information based on DSM‐5 or ICSD‐3 criteria in the NHANES database, prior investigations have similarly relied on self‐reported symptom questionnaires to ascertain insomnia status. Accordingly, the classification of insomnia in the current study was based on symptom‐derived criteria, rather than constituting a formal diagnosis according to DSM‐5 or ICSD‐3 standards. These questions mainly include: (1) “How often have trouble falling asleep (SLQ080)?” (2) “How often wake up during the night (SLQ090)?” (3) “How often wake up too early in the morning (SLQ100)?” If all three responses are “Never,” the respondent is considered non‐insomniac [44, 45]. If any response is “Yes,” respondents are further classified based on frequency as follows: Mild (responses are “Rarely (1 time a month)” or “Sometimes (2–4 times a month)”), Moderate (responses are “Often (5–15 times a month)”), or Severe (responses are “Almost always (16–30 times a month)”). If any of the three responses is classified as Severe, the respondent is considered to have severe insomnia. If all responses are classified as Mild, the respondent is considered to have mild sleep disturbance. When both Mild and Moderate are present, the respondent is classified as having moderate sleep disturbance.

### 2.5 Outcome Assessment

Participant vital status was confirmed by cross‐referencing with the National Death Index (NDI). All‐cause mortality includes deaths from any cause. Cardiovascular mortality was defined according to the codes I00–I09, I11, I13, I20–I51, or I60–I69, as documented in the National Death Index. This definition encompasses both cardiovascular disease (CVD) and cerebrovascular disease [46]. In the National Center for Health Statistics (NCHS) 2019 public‐use linked mortality files (Supporting Information S1), the codes for CVD mortality are represented as 001 and 005.

### 2.6 Statistical Analysis

We carried out our data analysis using R version 4.1.1. Due to the NHANES database's complex multi‐stage and stratified sampling methodology, we applied the appropriate weighting variables as outlined by the data provider's instructions. For the weighted statistical analysis, we used the “gtsummary” package in R. Continuous variables were first assessed for normality, which showed a deviation from normal distribution. As a result, we reported these skewed data using medians and the 25th and 75th percentiles and employed the Wilcoxon test for statistical examination. Categorical variables were presented through numerical values and proportions, with statistical analysis performed using the chi‐square test.

To assess the relationship between PNI exposure and the risk of all‐cause or cardiovascular mortality among individuals with insomnia, we employed a weighted Cox regression model. Hazard ratios (HR) and their 95% confidence intervals (CI) were computed as evaluation metrics. To account for the influence of covariates such as age and gender on the analysis, we utilized three distinct models. By adjusting for various covariates in each model, we re‐evaluated the relationship between PNI exposure and mortality risk. We then generated Kaplan–Meier (KM) curves to evaluate survival outcomes across different PNI quantiles. Subgroup analyses were conducted to determine whether the relationship between PNI exposure and mortality risk remained consistent within various subpopulations. Interaction analyses were performed to assess if PNI exposure is an independent factor influencing mortality risk. Receiver Operating Characteristic (ROC) curves were used to delineate the sensitivity and specificity of PNI in predicting mortality risk. Finally, we constructed Restricted Cubic Spline (RCS) curves to quantify the relationship between PNI and hazard ratios (HR). This analysis clarified how HR changes with varying levels of PNI and elucidated the impact of different PNI levels on the incidence of all‐cause and cardiovascular mortality. In all statistical analyses, a *p*‐value of less than 0.05 was considered indicative of a statistically significant difference.

## 3 Results

### 3.1 Demographics

This cohort study ultimately included a total of 3161 individuals with insomnia, of whom 546 were classified as having severe insomnia, representing a weighted proportion of 16%. Based on the severity of insomnia, these individuals were categorized into three groups. The three groups exhibited statistically significant differences in terms of gender, household income, educational attainment, tobacco exposure, and the presence of underlying conditions such as hypertension and diabetes. However, no statistically significant differences were observed among the groups in terms of age, race, or alcohol consumption (Table 1).

**Table 1:** Demographic and clinic characteristics according to individuals with sleep disorders.

		Sleep disorders level				
Characteristic	*N* a	Overall, *N* = 3161 (100%)^b^	Mildly, *N* = 1909 (61%)^b^	Moderately, *N* = 706 (23%)^b^	Severely, *N* = 546 (16%)^b^	*p* value^c^
Age (years)	3161	46 (34, 58)	46 (33, 57)	46 (36, 58)	47 (35, 60)	0.071
Gender	3161					0.004
Female		1688 (54%)	964 (50%)	408 (59%)	316 (60%)	
Male		1473 (46%)	945 (50%)	298 (41%)	230 (40%)	
Race	3161					0.500
Mexican American		500 (6%)	334 (7%)	91 (5%)	75 (5%)	
Other Hispanic		200 (3%)	110 (3%)	47 (3%)	43 (4%)	
Non‐Hispanic White		1728 (75%)	1021 (74%)	406 (77%)	301 (74%)	
Non‐Hispanic Black		594 (10%)	360 (9%)	131 (10%)	103 (10%)	
Other Race		139 (6%)	84 (6%)	31 (5%)	24 (6%)	
PIR	3161	3.27 (1.77, 5.00)	3.42 (1.90, 5.00)	3.37 (1.84, 5.00)	2.24 (1.22, 4.24)	<0.001
Education	3161					<0.001
< High school		796 (16%)	448 (14%)	164 (15%)	184 (23%)	
High school or some college		1686 (56%)	1002 (54%)	398 (59%)	286 (60%)	
College or above		679 (28%)	459 (32%)	144 (26%)	76 (16%)	
Smoking	3161					<0.001
No		1579 (51%)	1036 (56%)	331 (47%)	212 (36%)	
Yes		1582 (49%)	873 (44%)	375 (53%)	334 (64%)	
Drinking	3161					0.700
No		517 (13%)	319 (14%)	112 (13%)	86 (13%)	
Yes		2644 (87%)	1590 (86%)	594 (87%)	460 (87%)	
Hypertension	3161					<0.001
No		1904 (66%)	1222 (69%)	406 (64%)	276 (56%)	
Yes		1257 (34%)	687 (31%)	300 (36%)	270 (44%)	
Diabetes	3161					<0.001
No		2640 (89%)	1621 (90%)	599 (90%)	420 (82%)	
Yes		521 (11%)	288 (10%)	107 (10%)	126 (18%)	
Have trouble falling asleep	3161					<0.001
Never		784 (24%)	579 (29%)	123 (18%)	82 (13%)	
Mildly		1683 (54%)	1330 (71%)	265 (35%)	88 (14%)	
Moderately		390 (13%)	0 (0%)	318 (47%)	72 (13%)	
Severely		304 (9%)	0 (0%)	0 (0%)	304 (60%)	
Wake up during night	3161					<0.001
Never		572 (18%)	480 (25%)	47 (7%)	45 (8%)	
Mildly		1713 (55%)	1429 (75%)	222 (31%)	62 (11%)	
Moderately		529 (17%)	0 (0%)	437 (62%)	92 (16%)	
Severely		347 (10%)	0 (0%)	0 (0%)	347 (65%)	
Wake up too early in the morning	3161					<0.001
Never		877 (28%)	720 (38%)	94 (13%)	63 (12%)	
Mildly		1533 (49%)	1189 (62%)	249 (37%)	95 (19%)	
Moderately		461 (15%)	0 (0%)	363 (50%)	98 (19%)	
Severely		290 (8%)	0 (0%)	0 (0%)	290 (50%)	
OSA	3161					0.042
No		3019.00 (95.35%)	1846.00 (96.35%)	667.00 (94.29%)	506.00 (92.99%)	
Yes		142.00 (4.65%)	63.00 (3.65%)	39.00 (5.71%)	40.00 (7.01%)	
BMI (kg/m)	3161	27.40 (23.93, 31.71)	27.56 (23.91, 31.55)	27.16 (23.96, 31.40)	27.53 (23.97, 32.58)	0.400
Creatinine (mg/dL)	3161	0.89 (0.74, 1.00)	0.90 (0.76, 1.00)	0.82 (0.72, 1.00)	0.82 (0.70, 0.98)	0.013
Albumin (g/L)	3161	42.00 (40.00, 44.00)	43.00 (40.00, 45.00)	42.00 (40.00, 44.00)	42.00 (40.00, 44.00)	0.008
Lymphocyte count (×10^9^)	3161	1.90 (1.60, 2.30)	1.90 (1.50, 2.30)	1.90 (1.60, 2.30)	2.10 (1.70, 2.50)	<0.001
ALT (U/L)	3161	21.00 (17.00, 29.00)	22.00 (17.00, 29.00)	21.00 (17.00, 30.00)	21.00 (16.00, 28.00)	0.300
AST (U/L)	3161	23.00 (20.00, 27.00)	23.00 (20.00, 28.00)	23.00 (20.00, 27.00)	23.00 (19.00, 27.00)	0.300
Total bilirubin (umol/L)	3161	11.97 (10.26, 15.39)	13.68 (10.26, 17.10)	11.97 (10.26, 15.39)	11.97 (10.26, 15.39)	0.009
Triglycerides (mmol/L)	3161	1.28 (0.88, 1.86)	1.26 (0.87, 1.83)	1.25 (0.86, 1.81)	1.42 (0.95, 2.15)	0.031
Total cholesterol (mmol/L)	3161	5.02 (4.34, 5.74)	4.94 (4.32, 5.62)	5.09 (4.42, 5.87)	5.15 (4.34, 6.04)	0.009
Glycosylated hemoglobin (%)	3161	5.30 (5.10, 5.60)	5.30 (5.10, 5.60)	5.40 (5.10, 5.60)	5.40 (5.10, 5.70)	0.003
Fasting blood glucose (mmol/L)	3161	5.50 (5.11, 5.94)	5.50 (5.11, 5.88)	5.44 (5.07, 5.94)	5.55 (5.16, 6.00)	0.130
Cotinine (ng/mL)	3161	0.08 (0.02, 49.59)	0.06 (0.02, 2.24)	0.10 (0.03, 48.91)	0.72 (0.03, 223.91)	<0.001
SBP (mm Hg)	3161	118 (109, 129)	118 (110, 128)	118 (109, 130)	119 (110, 131)	0.400
DBP (mm Hg)	3161	70 (62, 77)	70 (62, 77)	70 (63, 78)	71 (62, 78)	0.300
PNI	3161	52.00 (49.50, 55.00)	52.00 (49.50, 55.00)	52.00 (49.00, 54.50)	53.00 (49.50, 55.50)	0.071

Abbreviations: ALT, alanine aminotransferase; AST, aspartate aminotransferase; BMI, body mass index; DBP, diastolic blood pressure; OSA, obstructive sleep apnea; PIR, ratio of family income to poverty; SBP, systolic blood pressure.

^a^ *N* not Missing (unweighted).

^b^ Median (IQR) for continuous; *n* (%) for categorical.

^c^ Wilcoxon rank‐sum test for complex survey samples; chi‐squared test with Rao and Scott's second‐order correction.

Additionally, individuals with severe sleep disturbances appeared to be those with higher levels of tobacco exposure but lower household income and educational attainment. These individuals also seemed to be more prone to having comorbidities such as hypertension and diabetes, and obstructive sleep apnea. Interestingly, individuals with severe insomnia had higher levels of triglycerides, cholesterol, and serum cotinine compared to those with mild insomnia.

### 3.2 PNI as a Protective Factor Against Cardiovascular and All‐Cause Mortality in Insomniacs

To elucidate the relationship between PNI exposure and the risk of cardiovascular and all‐cause mortality among individuals with insomnia, we employed a weighted Cox regression model and calculated hazard ratios (HR). In the weighted Cox regression analysis, the insomnia cohort was divided into four groups based on PNI quartiles: Q1 < 49.00 [reference], 49.00 ≤ Q2 < 52.00, 52.00 ≤ Q3 < 55.00, and Q4 ≥ 55.00 (Table 2).

**Table 2:** Multivariate Cox regression analysis of PNI and mortality.

	Characteristic	HR	95% CI	*p* value	*p* for trend	Characteristic	HR	95% CI	*p* value	*p* for trend
	PNI for CVD mortality					PNI for all‐cause mortality				
Model 1	Q1	Reference	—	—	<0.001	Q1	Reference	—	—	<0.001
	Q2	0.50	0.30, 0.81	0.005		Q2	0.57	0.44, 0.74	<0.001	
	Q3	0.33	0.21, 0.53	<0.001		Q3	0.46	0.34, 0.61	<0.001	
	Q4	0.24	0.14, 0.41	<0.001		Q4	0.37	0.27, 0.50	<0.001	
Model 2	Q1	Reference	—	—	<0.001	Q1	Reference	—	—	<0.001
	Q2	0.59	0.35, 0.99	0.044		Q2	0.63	0.49, 0.83	<0.001	
	Q3	0.50	0.32, 0.79	0.003		Q3	0.62	0.52, 0.74	<0.001	
	Q4	0.56	0.33, 0.94	0.029		Q4	0.77	0.61, 0.96	0.021	
Model 3	Q1	Reference	—	—	<0.001	Q1	Reference	—	—	<0.001
	Q2	0.60	0.35, 1.03	0.065		Q2	0.67	0.51, 0.88	0.004	
	Q3	0.44	0.29, 0.69	<0.001		Q3	0.56	0.48, 0.66	<0.001	
	Q4	0.52	0.29, 0.92	0.025		Q4	0.72	0.54, 0.96	0.025	

Abbreviations: CI, confidence interval; HR, hazard ratio.

Model 1: No confounding factors were adjusted.

Model 2: Adjusted for age, gender, race, education level, smoking status.

Model 3: Adjusted for age, gender, race, education level, hypertension, diabetes, creatinine, triglycerides, cotinine.

In Model 1, with no variable adjustments, individuals in the fourth quartile of PNI had approximately a 76% lower risk of cardiovascular mortality (Q4 vs. Q1, HR = 0.24) and about a 63% lower risk of all‐cause mortality (Q4 vs. Q1, HR = 0.37) compared to those in the first quartile. To account for potential confounding factors, we adjusted for various variables. In Model 2, we controlled for age, gender, race, educational attainment, and tobacco exposure. By adjusting for these variables, we isolated the relationship between PNI exposure and outcome events while minimizing the influence of other factors. We found that PNI remained a protective factor: individuals in the fourth quartile of PNI had approximately a 23% lower risk of all‐cause mortality and about a 44% lower risk of cardiovascular mortality compared to those in the first quartile. In Model 3, we further adjusted for laboratory results, including serum creatinine, triglycerides, serum cotinine levels, and the presence of comorbidities such as hypertension and diabetes. The protective effect of PNI exposure levels on outcome events remained significant. The regression analysis results indicated a negative correlation between PNI exposure and adverse outcome events (all‐cause and cardiovascular mortality), suggesting that an increase in PNI exposure within a certain range may act as a protective factor.

**Figure 2:**
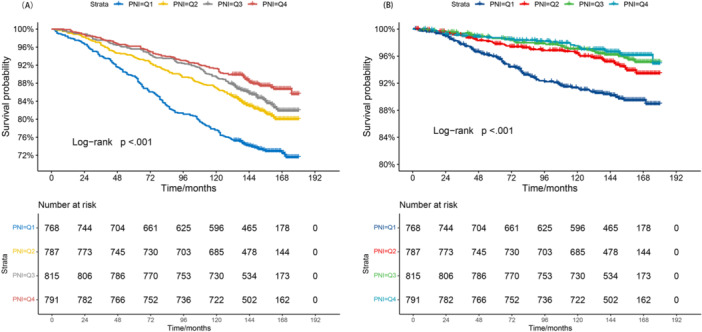
(A) Kaplan–Meier survival curve for all‐cause mortality. (B) Kaplan–Meier survival curve for cardiovascular mortality.

To further validate this conclusion, we generated Kaplan–Meier (KM) curves (Figure 2). After approximately 15 years of follow‐up, we found that individuals in the first quartile of PNI had fewer survivors compared to those in the fourth quartile, and this difference between the two groups was statistically significant (log‐rank *p* < 001).

### 3.3 Subgroup Analysis and Interaction Analysis

**Figure 3:**
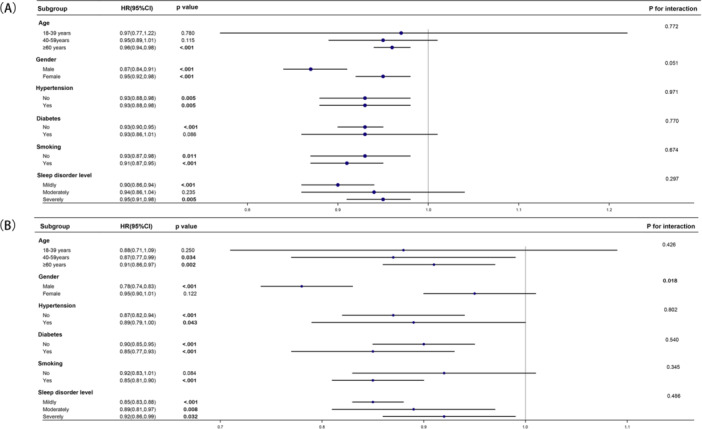
(A) Subgroup analysis for the association between PNI and all‐cause mortality. (B) Subgroup analysis for the association between PNI and cardiovascular mortality. Subgroup analyses were conducted using a weighted Cox regression model. Interaction analysis utilizes the Likelihood Ratio Test.

As previously mentioned, PNI is a protective factor for outcome events. To determine whether this protective effect remains consistent across different subgroups, we conducted a subgroup analysis (Figure 3). These subgroups primarily include age, gender, presence of hypertension and diabetes, tobacco exposure, and different levels of insomnia.

The forest plot results (Figure 3A) indicated that the protective effect of PNI exposure on all‐cause mortality remained significant among individuals over the age of 60 and those without diabetes. These populations included individuals of different genders, with and without hypertension, and those who do or do not smoke. However, we found that the protective effect of PNI exposure on all‐cause mortality appeared to be less pronounced in individuals with moderate insomnia. The protective effect of PNI exposure on cardiovascular mortality was more pronounced among smoking men over the age of 40. This group included individuals with or without hypertension and diabetes, as well as those with varying levels of insomnia (Figure 3B).

To evaluate whether the PNI is an independent protective factor, interaction analyses were performed. The protective effect of PNI on all‐cause mortality remained consistent across subgroups of age, sex, smoking status, hypertension, diabetes, and insomnia severity (all interaction *p*‐values > 0.05). In contrast, the protective effect of PNI on cardiovascular mortality was modified by sex (interaction *p* = 0.018), with the association being significantly stronger in men, resulting in lower cardiovascular mortality among males compared to females at comparable PNI levels.

### 3.4 Sensitivity and Specificity Analysis

**Figure 4:**
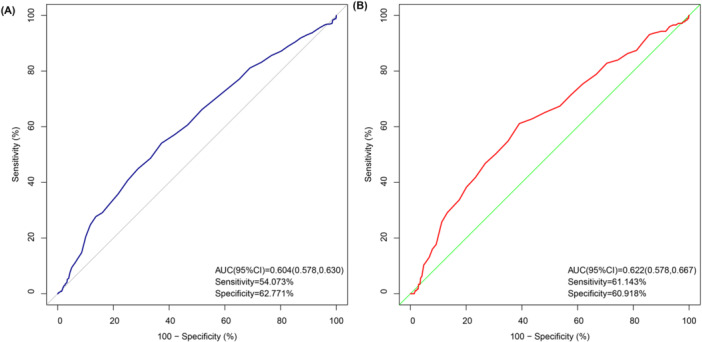
(A) ROC curve analysis of PNI and all‐cause mortality. (B) ROC curve analysis of PNI and cardiovascular mortality.

The construction of ROC curves further clarified the sensitivity and specificity of PNI in predicting outcome events (Figure 4). The Area Under the Curve (AUC) for PNI predicting all‐cause mortality was 0.604 (95% CI: 0.578, 0.630), while the AUC for predicting cardiovascular mortality was 0.622 (95% CI: 0.578, 0.667). PNI demonstrated better sensitivity for predicting cardiovascular mortality (sensitivity = 61.14%) compared to all‐cause mortality, whereas PNI showed higher specificity for predicting all‐cause mortality (specificity = 62.77%).

### 3.5 Quantitative Relationship Between PNI and Hazard Ratio: Restricted Cubic Splines Curve Analysis

**Figure 5:**
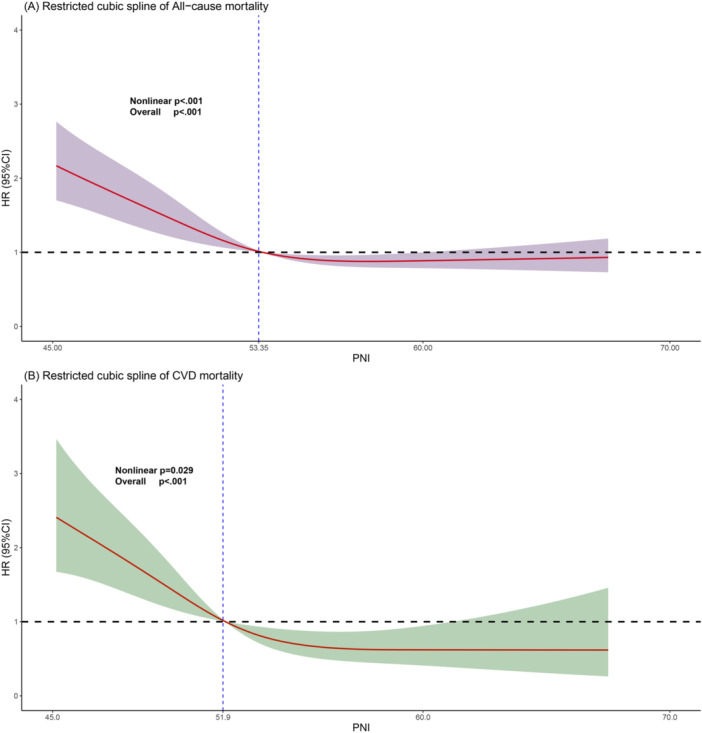
(A) Non‐linear relationship between PNI and all‐cause mortality. (B) Non‐linear relationship between PNI and cardiovascular mortality. Adjusted for age, gender, race, education, hypertension, diabetes, fasting blood glucose level, triglycerides, ALT, AST, cotinine. Likelihood ratio test was used in the restricted cubic spline regression.

If PNI is a protective factor for outcome events, what level of PNI is considered safe for individuals with insomnia? The Restricted Cubic Splines (RCS) curve analysis provides the answer. The creation of the RCS curve involves first calculating the Hazard Ratio (HR) values using weighted Cox regression analysis. Subsequently, the predicted HR values are modeled against PNI values, and then curve fitting is performed (Figure 5). We found that the relationship between PNI and the Hazard Ratios (HR) for both all‐cause mortality and cardiovascular mortality is non‐linear (with *p*‐values for non‐linearity all less than 0.05). There are statistically significant differences between the various segments in the plots (with overall *p*‐values all less than 0.05).

In Figure 5A, when PNI is below 53.35, the HR value decreases gradually with increasing PNI, and at PNI = 53.35, the HR value is exactly 1. Additionally, for PNI < 53.35, the HR and its 95% CI do not include values greater than 1 or less than 1, indicating significant statistical differences in this range. Within a certain range, as PNI continues to increase (PNI > 53.35), both the HR and its 95% CI remain below 1, which reaffirms that an increase in PNI reduces the risk of all‐cause mortality in individuals with insomnia within this range. However, beyond a certain threshold, as PNI continues to increase, the 95% CI for HR spans both above and below 1, suggesting no significant statistical differences in this region. Therefore, we conclude that PNI < 53.35 is a risk factor for all‐cause mortality in individuals with insomnia. In Figure 5B, a similar pattern is observed. When PNI < 51.9, the HR value for cardiovascular mortality is greater than 1. As PNI continues to increase, the HR value initially decreases but then levels off, showing minimal change. However, in this leveling‐off region, the 95% CI for HR spans both above and below 1, indicating no statistical significance. Hence, we infer that a PNI level below 51.9 is a risk factor for cardiovascular mortality among individuals with insomnia.

## 4 Discussion

Drawing on the multistage, complex, and stratified sampling design of the NHANES database, this cohort study uncovers the intricate link between the nutritional marker PNI and the occurrence of adverse outcomes, including all‐cause and cardiovascular mortality, in individuals suffering from insomnia. What makes this study particularly intriguing is that it represents the first report linking PNI with adverse outcomes in the insomnia population. This study utilized data that were subjected to strict quality control, with reliable and traceable sources, lending strong credibility to the findings. Distinct from conventional epidemiological research, the study employed weighted Cox regression analysis. This method improves the generalizability and representativeness of the findings, making the results more convincing despite the limited sample size. Our analyses indicate that the PNI serves as an independent protective factor against adverse events in individuals with insomnia. Notably, within specific limits, lower PNI levels are associated with a heightened risk of cardiovascular death and all‐cause mortality among this group. This finding indicates that during the clinical management of insomnia patients, attention should be paid to their PNI levels. Maintaining PNI levels above the critical value (with a minimum threshold of 51.90) could yield significant benefits for these individuals.

Insomnia affects 12%–20% of the general population, but this figure increases to 30% to 48% in older adults [47]. Previous research has often focused on factors such as age, gender, external stressors, use of electronic devices, and the consumption of stimulants like coffee, and their impact on insomnia [48]. Recently, researchers are paying more attention to nutritional status, as it has been recognized as a critical factor in determining patient outcomes [49, 50, 51]. Existing research evidence indicates a causal relationship between malnutrition and insomnia. Patients with severe insomnia have a 1.6‐fold increased risk of malnutrition compared to the general population. Additionally, a decline in Mini Nutritional Assessment (MNA) scores is associated with an increased risk of developing insomnia [52]. The Mini Nutritional Assessment Short Form (MNA‐SF), Long Form (MNA‐LF), Nutritional Risk Screening 2002 (NRS 2002), and Malnutrition Universal Screening Tool (MUST) are widely used tools for evaluating nutritional status [53, 54, 55, 56]. However, these indices can be complex to calculate, and their questionnaires are subjective, which can limit their accuracy and generalizability. Compared to the complex questionnaires and subjective assessments, the PNI is more objective and convenient. This is primarily because PNI is defined simply and can be easily calculated using laboratory results. There have been some studies on the use of PNI for assessing prognosis in cancer patients, kidney disease patients, and fracture patients [28, 29, 30, 31]. However, research on the relationship between PNI and insomnia is scarce. Our findings suggest that PNI holds potential research value for prognostic studies in populations with insomnia.

Based on the definition of PNI alone, factors influencing PNI levels include serum albumin levels and lymphocyte count. Serum albumin, as an important substance in the blood, plays roles in antioxidation, anti‐inflammation, maintaining plasma oncotic pressure, anticoagulation, and preventing platelet aggregation [57, 58, 59]. A study on insomnia caused by depression found that serum albumin levels were significantly reduced in individuals with insomnia. The authors suggested that this may be due to low protein levels impairing the body's ability to counteract oxidative stress [60]. First, low protein levels may reduce the synthesis of antioxidant enzymes, thereby weakening cellular antioxidant capacity. Key antioxidant enzymes such as glutathione peroxidase (GPX) and superoxide dismutase (SOD) play crucial roles in scavenging reactive oxygen species (ROS) [61]. Low protein levels may also impair mitochondrial function and exacerbate oxidative stress. As a major source of intracellular ROS, mitochondria can be further damaged through oxidative stress that affects mitochondrial DNA replication and maintenance [62]. Furthermore, low protein levels may amplify oxidative stress by influencing the endoplasmic reticulum (ER) stress pathway [63]. ER stress is closely associated with oxidative stress; studies show that oxidative stress can activate the PERK signaling pathway of ER stress, leading to insulin resistance. Thus, low protein levels may further compromise cellular antioxidant defenses by interfering with ER stress responses. Our study found that, after weighted analysis, serum albumin levels in individuals with severe insomnia were slightly lower than those in individuals with mild insomnia (Table 1, *p* = 0.008). It has been well established by most studies that a decrease in serum albumin levels can lead to the occurrence of cardiovascular adverse events [64, 65, 66]. Therefore, a decline in serum albumin levels is a risk factor for both insomnia and cardiovascular adverse events. A follow‐up study on percutaneous coronary intervention found that a decrease in lymphocyte count is a risk factor for poor prognosis and reduced 3‐year survival rates in patients with coronary artery disease [67]. However, there is limited research on whether lymphocyte count affects the prognosis of individuals with insomnia. A simultaneous decrease in serum albumin levels and lymphocyte count is a risk factor for cardiovascular adverse events. A decrease in both serum albumin levels and lymphocyte count also results in a lower PNI, which may explain why, within a certain range, a decrease in PNI levels is associated with an increased risk of cardiovascular mortality in individuals with insomnia.

There are numerous factors affecting insomnia patients, such as age, gender, smoking status, and the presence of comorbidities like hypertension and diabetes [1, 47, 48, 68, 69, 70, 71]. To account for the influence of these factors on the analysis, we adjusted for these covariates to ensure they were at similar levels before examining the relationship between PNI and outcome events. After adjusting for these variables, the protective effect of PNI on outcome events remained significant. We clarified the relationship between PNI and hazard ratio (HR) using restricted cubic spline (RCS) analysis. This analysis quantified the relationship between PNI and HR, enhancing its predictive value.

Our study has several advantages. Firstly, the data were sourced from the NHANES database, which is a large‐scale, complex, stratified sampling survey with rigorous research design and data collection protocols, ensuring the reliability of the results. Additionally, the data are publicly available and transparent, allowing other researchers to verify the findings. Secondly, the use of weighted analysis methods enhances the representativeness of the findings, even with a relatively small sample size. The study included 3161 individuals with insomnia, who held a weighted proportion in the original NHANES database. This weighting underscores their representation of the broader insomnia population across the United States from 2005 to 2008. Finally, we quantified the relationship between PNI and HR, clarifying the specific PNI levels at which it becomes a risk factor for adverse outcome events in individuals with insomnia. This enhances the practical applicability of the study. Nevertheless, our study has certain limitations. Firstly, the cross‐sectional nature of this study limited our ability to assess longitudinal changes in PNI levels among individuals with insomnia, as continuous monitoring data were not available. Secondly, we identified that a PNI below a specific threshold independently predicted cardiovascular and all‐cause mortality in insomnia patients. However, whether elevated PNI levels confer protection remains uncertain, potentially due to the relatively low number of adverse outcome events in the database. Further prospective cohort studies are needed to clarify this relationship. Finally, the limited number of cardiovascular events likely contributed to the suboptimal area under the receiver operating characteristic curve (AUC) values. Although our analysis adjusted for age, sex, hypertension, and diabetes, the inclusion of additional unmeasured confounders might also partially explain the modest discriminative performance observed. Due to missing data for certain variables in the NHANES database, complete patient information could not be obtained for all participants. Although we have appropriately handled these cases, we acknowledge that bias arising from missing information remains a concern. The present study represents a preliminary exploration, identifying the potential role and value of PNI in individuals with insomnia, and thereby providing a direction for future research. Nevertheless, before PNI can be translated into clinical practice, extensive cohort studies are still warranted for further validation. Only through rigorous validation can research findings ultimately inform clinical decision‐making.

## 5 Conclusion

In conclusion, this study identifies PNI as an independent prognostic biomarker associated with mortality risks in individuals with insomnia. While this association does not yet constitute evidence for direct clinical application, it highlights the importance of nutritional and inflammatory pathways in the long‐term health of these patients. Future research should focus on validating PNI's predictive utility in independent cohorts and exploring whether interventions aimed at optimizing PNI can translate into tangible survival benefits, before any recommendations for routine clinical management can be considered.

## Author Contributions

**Zihao Liu:** writing – original draft, methodology, writing – review and editing, formal analysis, data curation. **Linguo Gu:** writing – review and editing, methodology, supervision, writing – original draft.

## Funding

The authors have nothing to report.

## Ethics Statement

This study does not involve human or animal ethics, and all data is sourced from a public database that is publicly available and free of charge. Users do not need to apply for ethical approval again. This study utilized de‐identified data from the National Health and Nutrition Examination Survey (NHANES), a publicly available database maintained by the Centers for Disease Control and Prevention (CDC). The NHANES study protocol was reviewed and approved by the NCHS Research Ethics Review Board. Written informed consent was obtained from all NHANES participants. As this research involved secondary analysis of pre‐existing anonymized data, no direct patient or public involvement in the design, conduct, or reporting of the study was applicable. All original NHANES participants provided informed consent during primary data collection by the CDC. The findings of this study aim to contribute to public health knowledge by reusing open‐access data for evidence generation.

## Conflicts of Interest

The authors declare no conflicts of interest.

## Transparency Statement

Linguo Gu affirms that this manuscript is an honest, accurate, and transparent account of the study being reported; that no important aspects of the study have been omitted; and that any discrepancies from the study as planned (and, if relevant, registered) have been explained.

## Supporting information


Supporting File

## Data Availability

The data that supports the findings of this study are available in the Supporting material of this article.
